# Estimated Health Outcomes and Costs of COVID-19 Prophylaxis With Monoclonal Antibodies Among Unvaccinated Household Contacts in the US

**DOI:** 10.1001/jamanetworkopen.2022.8632

**Published:** 2022-04-22

**Authors:** Abraham D. Flaxman, Rodal Issema, Ruanne V. Barnabas, Jennifer M. Ross

**Affiliations:** 1 Institute for Health Metrics and Evaluation, University of Washington, Seattle; 2 Department of Epidemiology, University of Washington, Seattle; 3 International Clinical Research Center, Department of Global Health, University of Washington, Seattle; 4 Division of Infectious Diseases, Massachusetts General Hospital, Boston; 5 Harvard Medical School, Boston, Massachusetts; 6 Division of Allergy and Infectious Diseases, Department of Medicine, University of Washington, Seattle

## Abstract

**Importance:**

The COVID-19 pandemic has led to more than 900 000 deaths in the US and continues to disrupt lives even as effective vaccines are available.

**Objective:**

To estimate the health outcomes and net cost of implementing postexposure prophylaxis (PEP) with monoclonal antibodies (mAbs) against household exposure to COVID-19.

**Design, Setting, and Participants:**

This study is a decision analytical model of results from a randomized clinical trial of casirivimab with imdevimab administered as subcutaneous injections to unvaccinated, SARS-CoV-2–negative household contacts of people with confirmed COVID-19 with complementary data on household demographic structure, vaccine coverage, and confirmed COVID-19 case counts. The study used US data from May 2021 for a simulated population of US individuals of all ages within low-transmission or high-transmission scenarios.

**Exposures:**

Age, sex, race, ethnicity, and COVID-19 vaccination status.

**Main Outcome or Measures:**

Symptomatic infection, hospitalization, death, and net payer cost of monoclonal antibody PEP for COVID-19.

**Results:**

In a month of transmission intensity similar to that of May 2021, a mAb PEP program reaching 50% of exposed, unvaccinated household members aged 50 years and older was estimated to avert 1820 symptomatic infections (95% uncertainty interval [UI], 1220-2454 symptomatic infections), 528 hospitalizations (95% UI, 354-724 hospitalizations), and 84 deaths (95% UI, 55-116 deaths) in a low-transmission scenario and 4834 symptomatic infections (95% UI, 3375-6257 symptomatic infections), 1404 hospitalizations (95% UI, 974-1827 hospitalizations), and 223 deaths (95% UI, 152-299 deaths) in a high-transmission scenario. Without mAb PEP, the estimated cost of hospitalizations due to COVID-19 infections from household exposure in the lower transmission scenario was $149 million (95% UI, $115-$196 million), whereas the estimated hospitalization cost in the higher transmission scenario was $400 million (95% UI, $312-$508 million). In the lower transmission scenario, mAb PEP administered to 50% of eligible contacts aged 80 years and older was estimated to have 82% probability of saving costs, but was not associated with cost savings at age thresholds of 50 years and older or 20 years and older. In contrast, in the high-transmission scenario, mAb PEP administered to 50% of eligible household contacts had estimated cost savings in 100% of simulations at the 80-year age threshold, 96% of simulations at the 50-year threshold, and 2% of simulations at the 20-year thresholds.

**Conclusions and Relevance:**

In this modeling study of a simulated US population, a mAb PEP for COVID-19 program was estimated to improve health outcomes and reduce costs. In the setting of a susceptible variant of SARS-CoV-2, health system and public health actors would have an opportunity to improve health and reduce net payer costs through COVID-19 PEP with mAbs.

## Key Points

**Question:**

What are the potential health outcomes and costs of SARS-CoV-2 monoclonal antibody postexposure prophylaxis (PEP) for household contacts of people with COVID-19 in the US?

**Findings:**

In this decision analytical model study, for a month with transmission intensity similar to that of May 2021, a monoclonal antibody PEP program reaching 50% of exposed, unvaccinated household members aged 50 years and older was estimated to avert 528 hospitalizations and 84 deaths in a low-transmission scenario and 1404 hospitalizations and 223 deaths in a high-transmission scenario. The program was also estimated to be cost saving to payers in the high-transmission scenario as a result of averted hospitalizations.

**Meaning:**

These findings suggest that COVID-19 PEP with monoclonal antibodies may be associated with reduced costs and improved population health.

## Teaser

This decision analytical model study estimates the health outcomes and net cost of implementing postexposure prophylaxis with monoclonal antibodies (mAbs) against household exposure to COVID-19.

## Introduction

The COVID-19 pandemic, caused by SARS-CoV-2, has led to more than 900 000 deaths in the US and continues to disrupt lives even as effective vaccines are available.^1,2^ Initially, nonpharmaceutical interventions led to sustained declines in SARS-CoV-2 infections and COVID-19 deaths, but this was followed by multiple resurgences with the emergence of more transmissible SARS-CoV-2 variants of concern.^3,4^ Over time, prevention and treatment interventions for SARS-CoV-2 expanded dramatically from repurposed antivirals to highly effective vaccines and promising monoclonal antibodies (mAbs).^5,6,7^ Despite the success of COVID-19 vaccine development and initial distribution, the pace of vaccination slowed in the US, with a sizeable proportion of eligible persons remaining unvaccinated.^8^ Thus, observed COVID-19 cases, hospitalizations, and deaths have increased in multiple waves for several reasons, including vaccination coverage below the threshold for herd immunity, viral variants causing vaccine breakthrough infections, and declining immunity over time.^9,10^

In this setting of ongoing SARS-CoV-2 transmission, some mAb therapies are additional tools to prevent infection among unvaccinated individuals with a high-risk exposure to someone with SARS-CoV-2 infection.^11,12^ Antibody therapies are fast acting, since their ready-made antibodies can bind to antigen immediately, in contrast to vaccines that stimulate the body to produce an immune response over weeks.^13^ This property of fast-acting protection makes antibody therapies potentially attractive for use in household exposure situations where unvaccinated household contacts are at high risk of acquiring infection over a short time and can be identified rapidly.^14^ However, antibodies are costly to produce, which makes it important to assess their optimal use in health economic analyses.

A randomized clinical trial^11,15^ of the antibody combination of casirivimab with imdevimab (REGEN-COV, formerly known as REGN-COV2), conducted in early 2021, demonstrated efficacy in preventing symptomatic COVID-19 and polymerase chain reaction–positive SARS-CoV-2 infection (asymptomatic or symptomatic) when given to unvaccinated, SARS-CoV-2–negative household members of people with COVID-19 within 96 hours of their household member testing positive. The findings supported an emergency use authorization for use as postexposure prophylaxis (PEP) among people who are unvaccinated or unlikely to mount a protective response following vaccination and who are at high risk for progression to severe COVID-19.^11,15^ However, with continued viral evolution, REGEN-COV is not effective against the Omicron variant of SARS-CoV-2 that became dominant in early 2022, leading the US Food and Drug Administration to limit its use and exclude geographic regions where SARS-CoV-2 infections are likely due to nonsusceptible variants, although development of additional mAb therapies continues.^16,17,18^ In this decision analytical model study, we estimated the health outcomes and costs of a hypothetical PEP program in which unvaccinated household members who have been exposed to COVID-19 are given mAb PEP, to understand the potential public health significance of an approach that was shown to have strong clinical benefit against susceptible variants of SARS-CoV-2.

## Methods

### Study Design

We used a decision analytical model to combine results from the REGEN-COV randomized clinical trial with population data on household demographic structure, confirmed COVID-19 case counts and demographics, and vaccination coverage to estimate the number of symptomatic infections, hospitalizations, deaths, and net costs for mAb PEP programs of varying intensities.^11,19^ Our focus was on the general US population to provide evidence for public health policy. The key decision points in our model were the coverage level and age targeting of the intervention.

We report estimates according to the Consolidated Health Economic Evaluation Reporting Standards (CHEERS) reporting guideline.^20^ We published a replication archive for this analysis, which was coded in Python programming language version 3.8.8 (Python Software Foundation).^21^ These research activities used no identifiable private information and were therefore exempt from institutional board review, in accordance with 45 CFR §46.^22^

The baseline strategy in our analysis was to not implement mAb PEP, and we compared this with implementing mAb PEP at 4 intensities of coverage, where 25%, 50%, 75%, or 100% of unvaccinated individuals with household exposure to someone with confirmed COVID-19 and age above the minimum age threshold received mAb PEP. We explored 7 different age-based inclusion criteria (no minimum age for PEP and ages ≥20, ≥40, ≥50, ≥60, ≥70, and ≥80 years) and 2 scenarios of secondary attack rates. We used a time horizon corresponding to 1 wave of SARS-CoV-2 transmission, roughly 1 month, to evaluate the costs and outcomes associated with the program. We used a payer perspective to estimate net costs of the mAb PEP intervention and the (offset) costs of COVID-19 hospitalizations. We did not use time-discounting for costs or outcomes because of the short time horizon.

### Data Sources

Efficacy data come from the randomized clinical trial^11^ of the combination of mAbs casirivimab and indevimab administered as subcutaneous injections to unvaccinated, SARS-CoV-2–negative household contacts of people with confirmed COVID-19. The primary outcome of symptomatic SARS-CoV-2 infection occurred in 59 of 752 placebo recipients (7.8%) and 11 of 753 mAb PEP recipients (1.5%), indicating an 81% risk reduction.^11^ From this trial, we incorporated a household secondary attack rate of 7.8% in the low-transmission scenario. We developed a high-transmission scenario in which symptomatic SARS-CoV-2 infection developed in 21.1% of contacts who did not receive mAb PEP and 3.9% of mAb PEP recipients (81% risk reduction) using a meta-analysis of SARS-CoV-2 transmission in households.^23^ To quantify uncertainty, we sampled 100 attack rate values with and without mAb for each scenario.

We combined the effect size and secondary attack rates with data on the demographic structure of US households, national data on confirmed cases of COVID-19, and COVID-19 vaccine coverage and clustering by household. We used data from the American Community Survey to group individuals into households by age group, sex, and race and ethnicity and estimated the average number of individuals who would have a household exposure to COVID-19 from an index case in any demographic stratum.^19^ For example, to estimate the number of Black female individuals aged 80 years and older who would be exposed to a Black male individual aged 30 to 39 years with COVID-19, we identified all households in the American Community Survey data with a Black male individual aged 30 to 39 and counted the number of Black female individuals aged 80 years and older in each household. Then, we used the arithmetic mean of these counts as the estimate of the number of people exposed. We excluded individuals living in group quarters. To quantify uncertainty, we used nonparametric bootstrap resampling of 100 households with replacement for an index case in each demographic stratum.^24^

To find the age-specific, sex-specific, and race and ethnicity–specific rates of COVID-19 detection, we used confirmed COVID-19 case data from the Centers for Disease Control and Prevention for the month of May 2021.^25^ We calculated the fraction of cases in each demographic stratum using a complete-case analysis that dropped rows with missing data on age, sex, or race and ethnicity and then scaled these fractions to match the total count of cases including those with missing demographic data. We used nonparametric bootstrap resampling to quantify uncertainty.^24^

We modeled household clustering of COVID-19 vaccination status using the Kaiser Family Foundation survey from June 2021.^8^ In this nationally representative survey of US adults, 77% of vaccinated respondents indicated that everyone in their household also was vaccinated against COVID-19, and 69% of unvaccinated respondents reported that everyone in their household was unvaccinated. We used Bayes law to derive the fraction of confirmed cases who are unvaccinated from the population coverage and efficacy data and combined this with survey data on the percentage of unvaccinated people living in a household where everyone is unvaccinated (eAppendix in the Supplement).^26^ To quantify uncertainty, we sampled 100 values of this fraction.

We modeled the cost of COVID-19 hospitalization as $73 300 according to analysis by FAIR Health using *International Statistical Classification of Diseases and Related Health Problems, Tenth Revision *procedure codes.^27^ We included the unit cost of mAb PEP as $2100 on the basis of the federal government purchase price for REGEN-COV.^28^ We included the cost of mAb PEP administration as $450 according to the Centers for Medicare & Medicaid Services payment rates for administration of COVID-19 mAbs in a health care setting.^29^

### Statistical Analysis

#### Analytical Methods

**Figure 1.**
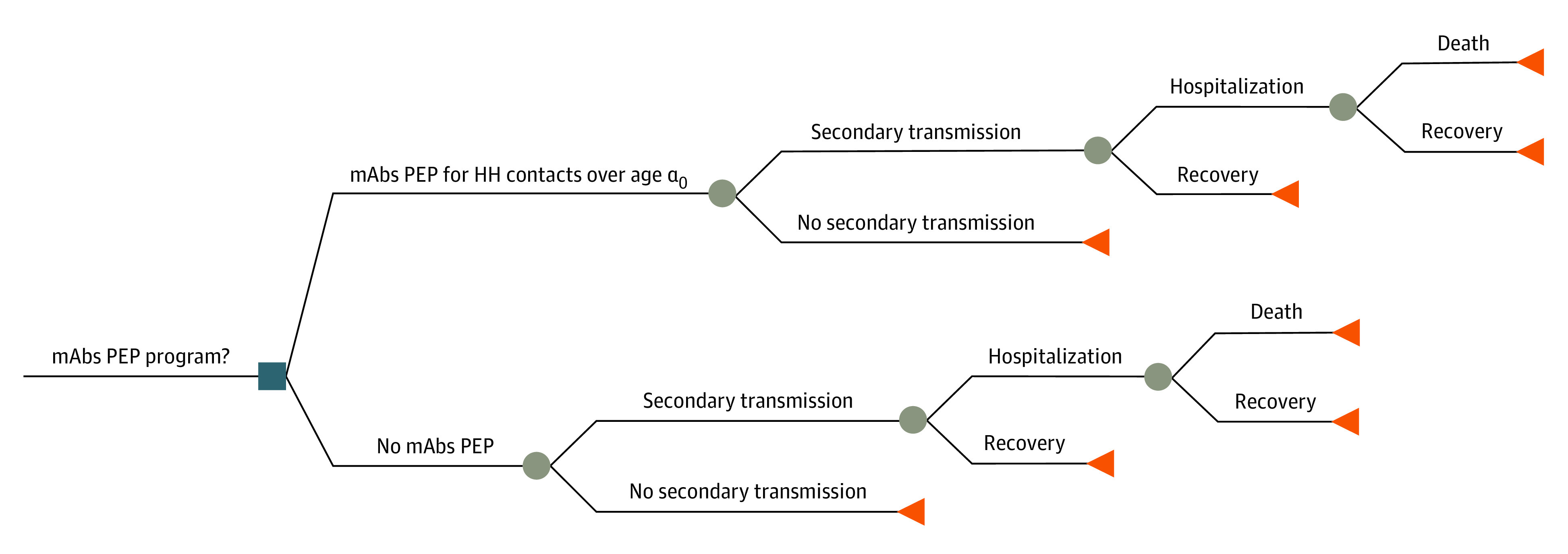
Decision Tree Representation of Analytical Model Diagram shows a single choice node (square) for postexposure prophylaxis (PEP) with monoclonal antibodies (mAbs) for each individual household (HH) contact, followed by a series of chance nodes (circles) for secondary infection, hospitalization, and mortality, leading eventually to terminal nodes (triangles) for recovery or death. α_0_ denotes age threshold.

To estimate the health outcomes and costs associated with the PEP program, we used an analytical model summarized by a decision tree (Figure 1) with a choice node for mAb PEP followed by chance nodes for symptomatic infection, hospitalization, and death. We used demographic data from confirmed cases of COVID-19 to identify households where PEP would be indicated and then used demographic data on household structure to identify the age, sex, and race or ethnicity of the household members who could receive mAb PEP. Details of the approach and parameter values are provided in the eAppendix in the Supplement. We used this approach to balance the complexity needed to capture the hypothesized differences between health outcomes by race and ethnicity with the simplicity of a multiplicative model structure. To investigate this hypothesis, we calculated the rates of infections, hospitalizations, and deaths averted, all stratified by race and ethnicity.

#### Model Assumptions

The model assumes a sequential progression of COVID-19 severity, with infection sometimes progressing to hospitalization, which sometimes progresses to death. We used infection rates measured in the REGEN-COV mAb PEP trial for the secondary attack rate in the first scenario and assumed that mAb PEP would have the same relative reduction in symptomatic SARS-CoV-2 infection in the second, higher transmission scenario. We calculated the rates of hospitalization and death by age, sex, and race and ethnicity from the Centers for Disease Control and Prevention case data. With this approach, when stratified by age, sex, and race and ethnicity, the fraction of symptomatic infections progressing to hospitalization (modeled) is the same as the fraction of confirmed cases progressing to hospitalization (observed). The average cost of hospitalization did not vary by individual characteristics. Also, after stratifying by age group, sex, and race and ethnicity, the household structure of people with confirmed cases COVID-19 was assumed to match that of the general population.

## Results

The REGEN-COV PEP trial^11,15^ included 1555 participants (753 in the treatment group and 752 in the placebo group), which we combined with confirmed cases data on 154 136 individuals, vaccine coverage survey data from 1888 individuals, and household structure data derived from 3 088 232 individuals in 1 276 716 households. We estimated that the 154 136 confirmed cases of COVID-19 in May 2021 in the US would result in at least 256 832 unvaccinated individuals (95% uncertainty interval [UI], 240 276-272 739 unvaccinated individuals) with household exposure to COVID-19. Using the racial and ethnic characteristics of households and COVID-19 cases described above, we estimated unvaccinated household contacts to include 43 219 (16.8%) non-Hispanic Black individuals, 50 380 (19.6%) Hispanic individuals, 135 800 (52.9%) non-Hispanic White individuals, and 27 433 (10.7%) members of other non-Hispanic racial groups (ie, American Indian/Alaska Native, Asian, multiple races, Native Hawaiian/other Pacific Islander, and any other race not specified). However, the race and ethnicity characteristics were missing for 32% of confirmed cases (49 986 individuals), 24% of hospitalizations (2186 individuals), and 20% of deaths (144 individuals), and our estimates rely on calculating the fraction of each demographic stratum with complete-case analysis.

In the first analysis scenario using the lower secondary attack rate observed in the REGEN-COV trial,^11,15^ providing PEP to 50% of unvaccinated household contacts aged 50 years and older was estimated to result in treatment of 28 309 individuals (95% UI, 25 961-30 330 individuals), with fewer individuals treated at higher age thresholds (Table 1). PEP coverage of 50% of unvaccinated contacts age 50 years and older was estimated to avert 1820 symptomatic COVID-19 cases (95% UI, 1220-2454 cases), 528 hospitalizations (95% UI, 354-724 hospitalizations), and 84 deaths (95% UI, 55-116 deaths). In the higher secondary attack rate scenario, providing PEP to 50% of unvaccinated household contacts aged 50 years and older was estimated to avert 4834 symptomatic infections (95% UI, 3375-6257 symptomatic infections), 1404 hospitalizations (95% UI, 974-1827 hospitalizations), and 223 deaths (95% UI, 152-299 deaths). Expanding the age threshold to 20 years and older was estimated to increase the averted burden, whereas restricting to age 80 years and older was estimated to reduce the averted burden (Table 1), with additional age thresholds in eTable 1 and eTable 2 in the Supplement. Expanding PEP coverage by half from 50% to 75% was estimated to result in corresponding 50% reductions in symptomatic COVID-19 cases, hospitalizations, and deaths.

**Table 1.** Monoclonal Antibody PEP Treatments Provided and COVID-19 Outcomes Estimated in Age Threshold Scenarios

Model	Household contacts, estimated No. (95% UI)		Secondary attack rate, %	Individuals treated with PEP, estimated No. (95% UI)	Outcomes, estimated No. (95% UI)		
	Total	Unvaccinated			Symptomatic COVID-19	Hospitalizations	Deaths
Baseline (no PEP)	381 357 (374 041-389 817)	256 832 (240 276-272 739)	7.8	0	20 124 (15 487-25 063)	2046 (1562-2516)	228 (173-284)
			21.1	0	53 847 (42 885-64 259)	5475 (4336-6551)	609 (468-763)
Age threshold for PEP (50% PEP coverage)							
≥80 y	4855 (4181-5706)	3271 (2730-3864)	7.8	1635 (1365-1932)	20 019 (15 422-24 928)	1978 (1516-2422)	204 (157-259)
			21.1	1635 (1365-1932)	53 567 (42 712-63 941)	5293 (4222-6341)	546 (424-672)
≥50 y	84 063 (80 884-88 099)	56 618 (51 923-60 660)	7.8	28 309 (25 961-30 330)	18 304 (14 252-22 669)	1518 (1201-1852)	144 (115-185)
			21.1	28 309 (25 961-30 330)	49 013 (39 885-58 779)	4071 (3328-4944)	386 (304-480)
≥20 y	234 381 (229 181-239 410)	157 853 (147 849-169 009)	7.8	78 926 (73 924-84 505)	15 053 (11 908-18 327)	1275 (1015-1578)	134 (107-174)
			21.1	78 926 (73 924-84 505)	40 377 (33 090-48 944)	3425 (2800-4178)	361 (283-452)

Abbreviations: PEP, postexposure prophylaxis; UI, uncertainty interval.

The estimated rates of averted COVID-19 cases, hospitalization, and death differed by race and ethnicity. Assuming 50% mAb PEP coverage of unvaccinated individuals aged 50 years and older, in the lower transmission scenario the estimated rates of averted secondary, symptomatic infections per 10 000 000 person-months were 80 among non-Hispanic Black individuals, 45 among Hispanic individuals, 55 among non-Hispanic White individuals, and 62 among members of other non-Hispanic racial and ethnic groups. In the higher transmission scenario, the corresponding rates of averted symptomatic infections per 10 000 000 person-months were 212 among non-Hispanic Black individuals, 118 among Hispanic individuals, 147 among non-Hispanic White individuals, and 164 among members of other non-Hispanic racial and ethnic groups. The estimated averted hospitalization rates per 10 000 000 person-months in the lower transmission scenario were 32 among non-Hispanic Black individuals, 15 among Hispanic individuals, 13 among non-Hispanic White individuals, and 22 among members of other non-Hispanic racial and ethnic groups, whereas the averted death rates per 10 000 000 person-months were 50 among non-Hispanic Black individuals, 30 among Hispanic individuals, 17 among non-Hispanic White individuals, and 49 among members of other non-Hispanic racial and ethnic groups. The estimated averted hospitalization rates per 10 000 000 person-months in the higher transmission scenario were 85 among non-Hispanic Black individuals, 39 among Hispanic individuals, 35 among non-Hispanic White individuals, and 57 among members of other non-Hispanic racial and ethnic groups, whereas the averted death rates per 10 000 000 person-months were 132 among non-Hispanic Black individuals, 81 among Hispanic individuals, 44 among non-Hispanic White individuals, and 131 among members of other non-Hispanic racial and ethnic groups.

**Figure 2.**
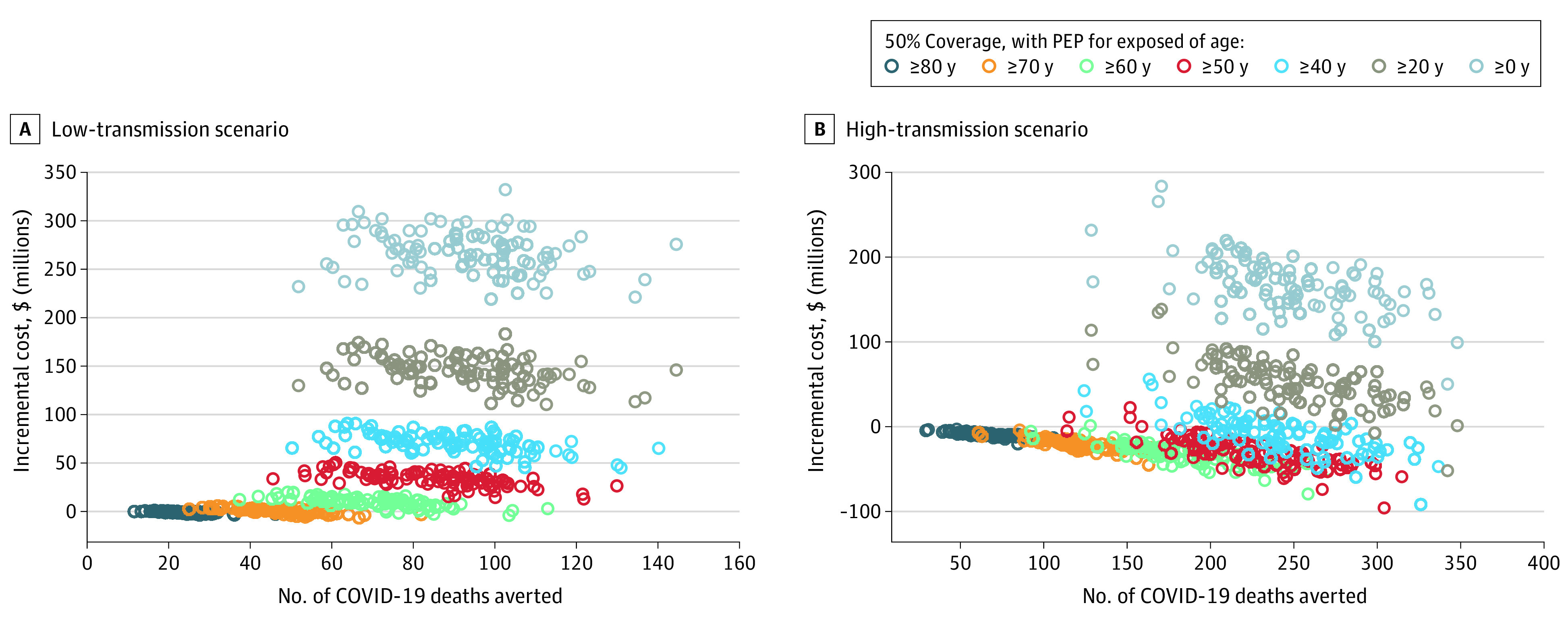
COVID-19 Deaths Averted by Age Threshold The number of deaths averted increases as the minimum age threshold for receiving postexposure prophylaxis (PEP) is decreased in the both low-transmission scenario (A) and high-transmission scenario (B). The incremental cost (including cost of PEP with monoclonal antibodies plus cost of COVID-19 hospitalizations) shows the trade-off between reducing hospitalization costs and increasing PEP costs.

Without mAb PEP, the estimated cost of hospitalizations due to COVID-19 infections from household exposure in the lower transmission scenario would be $149 million (95% UI, $115-$196 million), whereas estimated hospitalization costs in the higher transmission scenario would be $400 million (95% UI, $312-$508 million). The estimated costs of mAb PEP and hospitalization for different treatment age thresholds are shown in Table 2. In the lower transmission scenario, mAb PEP administered to 50% of eligible contacts aged 80 years and older was estimated to have 82% probability of saving costs, but was not associated with cost savings at age thresholds of 50 years and older or 20 years and older. In contrast, in the higher transmission scenario, mAb PEP administered to 50% of eligible household contacts had estimated cost-savings in 100% of simulations at the 80-year age threshold, 96% of simulations at the 50-year threshold, and 2% of simulations at the 20-year threshold. Figure 2 compares estimated deaths averted and incremental costs for additional age thresholds.

**Table 2.** Costs of Monoclonal Antibody PEP and Hospitalizations Under Different Age Threshold and Coverage Scenarios

Age threshold for PEP and PEP coverage rate	Secondary attack rate, %	Estimated costs (95% UI), $US million			
		PEP	Hospitalization	Total	Incremental
≥80 y					
50%	7.8	4 (3 to 5)	144 (112 to 189)	148 (115 to 194)	−1 (−3 to −1)
	21.1		386 (300 to 492)	390 (305 to 496)	−9 (−13 to −5)
75%	7.8	6 (5 to 8)	142 (110 to 185)	148 (115 to 192)	−1 (−4 to −1)
	21.1		380 (294 to 484)	386 (301 to 490)	−14 (−20 to −7)
100%	7.8	8 (7 to 10)	139 (108 to 182)	147 (116 to 191)	−2 (−5 to −2)
	21.1		373 (288 to 477)	381 (297 to 485)	−18 (−26 to −9)
≥50 y					
50%	7.8	72 (61 to 80)	111 (88 to 146)	183 (151 to 224)	34 (16 to −46)
	21.1		297 (223 to 378)	370 (295 to 452)	−30 (−60 to −6)
75%	7.8	108 (92 to 120)	91 (73 to 123)	200 (167 to 241)	51 (24 to −70)
	21.1		246 (183 to 315)	355 (288 to 422)	−45 (−90 to −9)
100%	7.8	145 (123 to 160)	72 (57 to 99)	217 (186 to 254)	68 (32 to −93)
	21.10		195 (141 to 250)	340 (278 to 400)	−60 (−120 to −11)
≥20 y					
50%	7.8	202 (175 to 222)	93 (74 to 125)	295 (254 to 343)	145 (114 to −172)
	21.1		250 (184 to 318)	452 (381 to 524)	52 (1 to 104)
75%	7.8	302 (262 to 334)	65 (51 to 89)	367 (319 to 417)	218 (171 to −258)
	21.1		176 (122 to 229)	478 (413 to 544)	78 (2 to 156)
100%	7.8	403 (350 to 445)	37 (23 to 62)	440 (385 to 492)	291 (228 to −344)
	21.1		101 (56 to 162)	504 (440 to 584)	105 (3 to 208)

Abbreviations: PEP, postexposure prophylaxis; UI, uncertainty interval.

## Discussion

In this decision analytical model study, we estimated that providing mAb PEP to unvaccinated household contacts of persons with COVID-19 aged 50 years and older in the setting of a susceptible SARS-CoV-2 variant would prevent COVID-19 disease and deaths and be cost-saving in the higher transmission scenario as a result of averted costs of hospitalization over 1 month of implementation in the US. In contrast, the lower transmission scenario observed in the REGEN-COV trial was unlikely to be cost-saving at an age threshold of 50 years and older, but was estimated to have an 82% probability of cost-savings at a threshold of age 80 years and older. We did not estimate COVID-19 cases and costs that could occur from a subsequent round of transmission from household contacts to other individuals. Despite this conservative assumption, the use of mAb PEP was estimated to avert morbidity and mortality and to be an efficient use of resources in the higher transmission scenario.

Early in 2022, the Omicron variant of SARS-CoV-2 became the dominant strain in the US, and administration of REGEN-COV was largely halted for lack of efficacy against this variant.^17^ Although there are many unknowns about the future course of the COVID-19 pandemic, mAb PEP could have a beneficial role in COVID-19 combination prevention for several reasons. First, less-than-full vaccination coverage among the US population has left persistent protection gaps, and household clustering of vaccination status means that unvaccinated people who are at greater risk for developing SARS-CoV-2 are more likely to share a household with other unvaccinated people. Second, mAb PEP may be acceptable to an unvaccinated person with a high-risk household exposure because it offers rapid protection compared with initiating a multiweek vaccine series and could be offered with follow-up vaccination. Third, promising advances in efforts to isolate and develop mAbs with activity against a broad range of sarbecoviruses could lessen the risk of loss of activity with future SARS-CoV-2 genotypes/variants.^18^ Finally, beyond households, mAb PEP may be used in other settings with high-risk exposures, such as long-term care facilities or among immunocompromised people who are less likely to mount an immune response to vaccination.^30^

Demonstrating the potential population health benefit and economic value of mAb PEP is crucial to overcoming the logistical challenges of implementing a novel intervention and advocating for production of sufficient antibody supply. Shortly following the emergency use authorization for REGEN-COV, the National Institutes of Health published guidelines prioritizing the use of mAb therapies for treatment of COVID-19 ahead of PEP when necessary to triage the use of mAb because of logistical or supply constraints.^31^ Methodological strengths of our analysis include the use of large, publicly available data sets on household composition, COVID-19 cases, and vaccine coverage; inclusion of high-quality efficacy data observed in a randomized clinical trial; the assumption that the health economic profile is insensitive to epidemic size because costs and outcomes scale with cases; and incorporation of uncertainty measures throughout the model.

### Limitations

This analysis also has limitations. First, the analysis took a payer perspective in focusing on the cost of the medication and administration without including the cost of building up the public health infrastructure needed to identify and notify eligible contacts. In addition, we did not identify data to compare the actual cost to health facilities of administering mAb PEP with the $450 per infusion that Centers for Medicare & Medicaid Services reimburses. Second, population structure may have changed as a result of social distancing. Third, there were missing data for confirmed cases, especially about race and ethnicity, hospitalizations, and deaths. Fourth, we modeled the whole US population without differentiating regionally for vaccine coverage, household composition, and COVID-19 case activity. Fifth, we did not analyze mAb PEP for people who were vaccinated but unlikely to mount a response as a result of immunosuppression because of a lack of data. Similarly, we did not distinguish within age groups for people with comorbidities that are associated with increased risk of COVID-19 progression or costs of hospitalization. Sixth, we assumed that unvaccinated individuals are susceptible to SARS-CoV-2 infection without accounting for partial immunity from prior SARS-CoV-2 infection.

## Conclusions

In this modeling study of a simulated US population, a mAb PEP for COVID-19 program was estimated to improve health outcomes and reduce costs. In the scenario of a susceptible SARS-CoV-2 variant, health system and public health actors in the US may have an opportunity to improve health and reduce costs through COVID-19 PEP with mAbs.

## Notes

Supplement. **eAppendix.** Supplemental Methods **eTable 1.** Health Outcomes of mAb PEP Programs by Age Threshold and Coverage Level in Low Attack Rate Scenario **eTable 2.** Health Outcomes of mAb PEP Programs by Age Threshold and Coverage Level in High Attack Rate Scenario
eReferences
