# Supplementary material for: Estimated Health Outcomes and Costs of COVID-19 Prophylaxis With Monoclonal Antibodies Among Unvaccinated Household Contacts in the US
Source: JAMA Netw Open. 2022 Apr 22;5(4):e228632. doi: 10.1001/jamanetworkopen.2022.8632 (PMC9034404; doi:10.1001/jamanetworkopen.2022.8632)
Supplement: Supplement. — eAppendix. Supplemental Methods eTable 1. Health Outcomes of mAb PEP Programs by Age Threshold and Coverage Level in Low Attack Rate Scenario eTable 2. Health Outcomes of mAb PEP Programs by Age Threshold and Coverage Level in High Attack Rate Scenario eReferences [file jamanetwopen-e228632-s001.pdf]

Supplemental Online Content

Flaxman AD, Issema R, Barnabas RV, Ross JM. Estimated health outcomes and costs of COVID-19 prophylaxis with monoclonal antibodies among unvaccinated household contacts in the US. *JAMA Netw Open*. 2022;5(4):e228632. doi:10.1001/jamanetworkopen.2022.8632

eAppendix. Supplemental Methods

eTable 1. Health Outcomes of mAb PEP Programs by Age Threshold and Coverage Level in Low Attack Rate Scenario

eTable 2. Health Outcomes of mAb PEP Programs by Age Threshold and Coverage Level in High Attack Rate Scenario

eReferences

This supplemental material has been provided by the authors to give readers additional information about their work.

© 2022 Flaxman AD et al. *JAMA Network Open.*

eAppendix. Supplemental Methods

Analytical steps:

1. Estimate the number of COVID-19 case count, n_1, and the age-/sex-/race-stratified case counts as
$$n_1(a,s,r) = \#\text{ cases for strata } (a,s,r).$$

2. Estimate the stratified number of unvaccinated household contacts who might benefit from mAbs PEP as
$$n_2(a,s,r) = \sum_{a',s',r'} p_{\text{unvax}} \times n_1(a',s',r') \times n_{hh}^{a',s',r'}(a,s,r),$$

   where $n_{hh}^{a',s',r'}(a,s,r)$ denotes the mean number of *other* people in strata (a,s,r) who live in a household with a person in strata (a',s',r') and p_{unvax} denotes the probability that a COVID-19 case occurs in a completely unvaccinated household.

3. Calculate the probability that a COVID-19 case occurs in a completely unvaccinated household as:
   - Equation 1:

   Pr(CC)=(Pr(CC|vax)×Pr(vax))+(Pr(CC|unvax)×Pr(unvax))

   - Equation 2:

   Pr(unvax|CC)=Pr(CC|unvax)×Pr(unvax)Pr(CC)

   - Equation 3:

   Pr(CC|vax)=(1−efficacy)×Pr(CC|unvax)

   - Substitute Equation 3 into Equation 1:

   Pr(CC)=((1−efficacy)×Pr(CC|unvax)×Pr(vax))+(Pr(CC|unvax)×Pr(unvax))

   =Pr(CC|unvax)(((1−efficacy) ×Pr(vax))+Pr(unvax))

   - Substitute Equation 1 into Equation 2:

   Pr(unvax|CC)=Pr(CC|unvax)×Pr(unvax)Pr(CC|unvax)(((1−efficacy) ×Pr(vax))+Pr(unvax))

   =Pr(unvax)((1−efficacy) ×Pr(vax))+Pr(unvax)

4. Estimate the number of people who receive PEP in scenarios with a range of coverage levels c and minimum age a_0 for receiving PEP as
$$n_{\text{PEP}}^{c,a_0}(a,s,r) = c \times n_2(a,s,r) \times [a \geq a_0],$$
   where $[a \geq a_0]$ is equal to 1 if $a \geq a_0$ and 0 otherwise.
5. Estimate the number of people who develop a symptomatic COVID-19 infection in each scenario as
$$n_{\text{COVID}}^{c,a_0}(a,s,r) = \text{ar}_{\overline{\text{PEP}}} \times \left(n_2(a,s,r) - n_{\text{PEP}}^{c,a_0}(a,s,r)\right) + \text{ar}_{\text{PEP}} \times n_{\text{PEP}}^{c,a_0}(a,s,r),$$
   where $\text{ar}_{\overline{\text{PEP}}}$ is the secondary attack rate without mAbs and ar_{PEP} is the secondary attack rate with mAbs.
6. Estimate the number of hospitalizations in each scenario as
$$n_{\text{hosp}}^{c,a_0}(a,s,r) = \text{hr}(a,s,r) \times n_{\text{COVID}}^{c,a_0}(a,s,r),$$
   where $\text{hr}(a,s,r)$ is the hospitalization rate for strata (a,s,r).
7. Estimate the number of deaths in each scenario as
$$n_{\text{death}}^{c,a_0}(a,s,r) = \text{hfr}(a,s,r) \times n_{\text{hosp}}^{c,a_0}(a,s,r),$$
   where $\text{hfr}(a,s,r)$ is the hospitalization fatality ratio for strata (a,s,r).
8. Estimate the cost of administering mAbs and the cost of COVID-19 hospitalizations as

© 2022 Flaxman AD et al. *JAMA Network Open.*

$$c_{\mathrm{mAbs}}^{c,a_0} = uc_{\mathrm{mAbs}} \times \sum_{a,s,r} n_{\mathrm{PEP}}^{c,a_0}(a,s,r),$$

$$c_{\mathrm{hosp}}^{c,a_0} = uc_{\mathrm{hosp}} \times \sum_{a,s,r} n_{\mathrm{hosp}}^{c,a_0}(a,s,r),$$

where uc_{mAbs} and uc_{hosp} are the unit costs for mAbs and COVID-19 hospitalization.

Parameters, values, data sources, and analytical methods:

- $n_1(a,s,r)$ comes from CDC confirmed cases; to deal with missing data, we computed the fraction of cases by age, sex, and race/ethnicity from the rows with complete data (complete-case analysis) and then scaled this value to the total number of cases detected during May 2021. We selected age groups to match those available in the CDC data, and collapsed race/ethnicity to white, Black, Hispanic, and all other race/ethnicities.[1]
- $n_{hh}^{a',s',r'}(a,s,r)$ comes from ACS PUMS data, which includes age in years, sex, race/ethnicity, and household ID.[2]
- We examined scenarios corresponding to all combinations of the age thresholds matched to the age groups from CDC data, and coverage levels of 0, 25%, 50%, 75%, and 100%.
- The secondary attack rates within households without PEP $\mathrm{ar}_{\overline{\mathrm{PEP}}} = 0.078$ and with PEP $\mathrm{ar}_{\mathrm{PEP}} = 0.014$ came from the cas/imdev RCT in the low attack rate scenario.[3] The attack rate without PEP in the high attack rate scenario $\mathrm{ar}_{\overline{\mathrm{PEP}}} = 0.211$ came from the meta-analysis by Thompson, et al.[4] We calculated the attack rate with PEP in the high attack rate scenario $\mathrm{ar}_{\mathrm{PEP}} = 0.038$ by applying the 81% relative risk reduction observed in the cas/imdev RCT.
- We calculated $\mathrm{hr}(a,s,r)$ from CDC data. We dropped all cases where age, sex, race/ethnicity, or hospitalization status was unknown.[1]
- We calculated $\mathrm{hfr}(a,s,r)$ similarly from CDC data, by dropping all cases which were not hospitalized or where age, sex, race/ethnicity, or mortality status was unknown.
- We determined the unit costs $uc_{\mathrm{mAbs}} = \$2{,}550$ using $2,100 as the cost of the medication based on the federal government purchase price and $450 as the cost of administration based on Centers for Medicare and Medicaid Services reimbursement rates.[5,6] We estimated the unit cost of hospitalization $uc_{\mathrm{hosp}} = \$73{,}300$ from recent literature.[7]

eTable 1: Health Outcomes of mAb PEP Programs by Age Threshold and Coverage Level in Low Attack Rate Scenario

Outcome	Age Threshold	Program Coverage				
		0%	25%	50%	75%	100%
**Number Treated with PEP**	**0**	0 (0-0)	64,208 (60,069-68,185)	128,416 (120,138-136,369)	192,624 (180,207-204,554)	256,832 (240,276-272,739)
	**20**	0 (0-0)	39,463 (36,962-42,252)	78,926 (73,924-84,505)	118,389 (110,886-126,757)	157,853 (147,849-169,009)
	**40**	0 (0-0)	23,029 (21,363-24,499)	46,058 (42,725-48,998)	69,086 (64,088-73,497)	92,115 (85,451-97,996)
	**50**	0 (0-0)	14,155 (12,981-15,165)	28,309 (25,961-30,330)	42,464 (38,942-45,495)	56,618 (51,923-60,660)
	**60**	0 (0-0)	7,048 (6,385-7,683)	14,095 (12,770-15,367)	21,143 (19,155-23,050)	28,191 (25,540-30,733)
	**70**	0 (0-0)	2,793 (2,467-3,137)	5,586 (4,934-6,275)	8,378 (7,400-9,412)	11,171 (9,867-12,549)
	**80**	0 (0-0)	818 (682-966)	1,635 (1,365-1,932)	2,453 (2,047-2,898)	3,271 (2,730-3,864)
**Symptomatic COVID-19 from household exposure**	**0**	20,124 (15,487-25,063)	15,999 (12,598-19,548)	11,874 (9,273-14,630)	7,750 (6,167-10,154)	3,625 (1,687-6,128)
	**20**	20,124 (15,487-25,063)	17,589 (13,695-21,695)	15,053 (11,908-18,327)	12,518 (9,862-15,451)	9,982 (7,843-12,586)
	**40**	20,124 (15,487-25,063)	18,644 (14,482-23,113)	17,164 (13,420-21,163)	15,684 (12,382-19,212)	14,204 (11,301-17,323)
	**50**	20,124 (15,487-25,063)	19,214 (14,885-23,866)	18,304 (14,252-22,669)	17,394 (13,590-21,471)	16,484 (12,964-20,274)
	**60**	20,124 (15,487-25,063)	19,671 (15,190-24,469)	19,218 (14,887-23,876)	18,765 (14,573-23,283)	18,313 (14,240-22,689)
	**70**	20,124 (15,487-25,063)	19,945 (15,371-24,832)	19,765 (15,256-24,600)	19,586 (15,132-24,369)	19,407 (15,005-24,138)
	**80**	20,124 (15,487-25,063)	20,072 (15,454-24,995)	20,019 (15,422-24,928)	19,967 (15,390-24,861)	19,914 (15,358-24,793)
**Hospitalizations from household exposure**	**0**	2,046 (1,562-2,516)	1,627 (1,278-1,972)	1,207 (955-1,492)	788 (628-1,034)	368 (171-625)
	**20**	2,046 (1,562-2,516)	1,660 (1,300-2,011)	1,275 (1,015-1,578)	889 (711-1,150)	503 (318-757)
	**40**	2,046 (1,562-2,516)	1,720 (1,339-2,078)	1,395 (1,113-1,719)	1,069 (843-1,348)	743 (581-1,002)
	**50**	2,046 (1,562-2,516)	1,782 (1,378-2,154)	1,518 (1,201-1,852)	1,253 (987-1,556)	989 (779-1,254)
	**60**	2,046 (1,562-2,516)	1,866 (1,437-2,272)	1,686 (1,314-2,043)	1,506 (1,188-1,850)	1,326 (1,041-1,643)
	**70**	2,046 (1,562-2,516)	1,953 (1,495-2,388)	1,860 (1,429-2,261)	1,767 (1,367-2,136)	1,674 (1,303-2,028)
	**80**	2,046 (1,562-2,516)	2,012 (1,538-2,469)	1,978 (1,516-2,422)	1,944 (1,493-2,375)	1,909 (1,471-2,329)

© 2022 Flaxman AD et al. *JAMA Network Open.*

**Deaths from household exposure**	**0**	228 (173-284)	181 (142-231)	134 (107-174)	88 (69-121)	41 (19-74)
	**20**	228 (173-284)	181 (142-231)	134 (107-174)	88 (69-121)	41 (19-74)
	**40**	228 (173-284)	182 (143-233)	137 (110-178)	92 (73-125)	47 (24-79)
	**50**	228 (173-284)	186 (145-236)	144 (115-185)	102 (80-134)	60 (34-90)
	**60**	228 (173-284)	192 (149-244)	157 (123-201)	122 (94-155)	87 (62-115)
	**70**	228 (173-284)	204 (157-258)	180 (141-233)	157 (123-206)	133 (104-174)
	**80**	228 (173-284)	216 (165-271)	204 (157-259)	192 (149-247)	180 (140-233)

© 2022 Flaxman AD et al. *JAMA Network Open.*

eTable 2: Health Outcomes of mAb PEP Programs by Age Threshold and Coverage Level in High Attack Rate Scenario

Outcome	Age Threshold	Program Coverage				
		0%	25%	50%	75%	100%
**Number Treated with PEP**	**0**	0 (0-0)	64,208 (60,069-68,185)	128,416 (120,138-136,369)	192,624 (180,207-204,554)	256,832 (240,276–272,739)
	**20**	0 (0-0)	39,463 (36,962-42,252)	78,926 (73,924-84,505)	118,389 (110,886-126,757)	157,853 (147,849-169,009)
	**40**	0 (0-0)	23,029 (21,363-24,499)	46,058 (42,725-48,998)	69,086 (64,088-73,497)	92,115 (85,451-97,996)
	**50**	0 (0-0)	14,155 (12,981-15,165)	28,309 (25,961-30,330)	42,464 (38,942-45,495)	56,618 (51,923-60,660)
	**60**	0 (0-0)	7,048 (6,385-7,683)	14,095 (12,770-15,367)	21,143 (19,155-23,050)	28,191 (25,540-30,733)
	**70**	0 (0-0)	2,793 (2,467-3,137)	5,586 (4,934-6,275)	8,378 (7,400-9,412)	11,171 (9,867-12,549)
	**80**	0 (0-0)	818 (682-966)	1,635 (1,365-1,932)	2,453 (2,047-2,898)	3,271 (2,730-3,864)
**Symptomatic COVID-19 from household exposure**	**0**	53,847 (42,885-64,259)	42,889 (35,175-51,613)	31,930 (26,007-39,412)	20,972 (15,719-26,847)	10,014 (4,374-18,798)
	**20**	53,847 (42,885-64,259)	47,112 (38,610-56,482)	40,377 (33,090-48,944)	33,643 (27,571-41,438)	26,908 (21,430-33,960)
	**40**	53,847 (42,885-64,259)	49,915 (40,369-59,755)	45,983 (37,772-55,254)	42,051 (34,466-50,770)	38,119 (31,476-46,592)
	**50**	53,847 (42,885-64,259)	51,430 (41,329-61,519)	49,013 (39,885-58,779)	46,596 (38,281-56,039)	44,178 (36,248-53,303)
	**60**	53,847 (42,885-64,259)	52,643 (42,107-62,862)	51,440 (41,329-61,466)	50,237 (40,552-60,070)	49,033 (39,892-58,673)
	**70**	53,847 (42,885-64,259)	53,369 (42,579-63,696)	52,892 (42,273-63,134)	52,414 (41,967-62,572)	51,937 (41,661-62,010)
	**80**	53,847 (42,885-64,259)	53,707 (42,799-64,100)	53,567 (42,712-63,941)	53,427 (42,626-63,783)	53,288 (42,539-63,624)
**Hospitalizations from household exposure**	**0**	5,475 (4,336-6,551)	4,360 (3,581-5,261)	3,246 (2,644-3,962)	2,132 (1,595-2,718)	1,017 (444-1,899)
	**20**	5,475 (4,336-6,551)	4,450 (3,654-5,367)	3,425 (2,800-4,178)	2,401 (1,864-3,015)	1,376 (840-2,091)
	**40**	5,475 (4,336-6,551)	4,609 (3,777-5,558)	3,744 (3,051-4,575)	2,878 (2,318-3,588)	2,013 (1,491-2,612)
	**50**	5,475 (4,336-6,551)	4,773 (3,885-5,747)	4,071 (3,328-4,944)	3,369 (2,751-4,147)	2,667 (2,145-3,369)
	**60**	5,475 (4,336-6,551)	4,996 (4,013-5,989)	4,518 (3,710-5,428)	4,039 (3,300-4,878)	3,561 (2,914-4,334)
	**70**	5,475 (4,336-6,551)	5,227 (4,170-6,258)	4,979 (4,003-5,964)	4,732 (3,859-5,672)	4,484 (3,708-5,380)
	**80**	5,475 (4,336-6,551)	5,384 (4,279-6,446)	5,293 (4,222-6,341)	5,202 (4,165-6,236)	5,111 (4,107-6,131)
	**0**	609 (468-763)	485 (383-604)	361 (283-452)	237 (176-311)	113 (48-202

© 2022 Flaxman AD et al. *JAMA Network Open.*

**Deaths from household exposure**	**20**	609 (468-763)	485 (383-604)	361 (283-452)	237 (176-311)	113 (48-202)
	**40**	609 (468-763)	490 (385-609)	370 (291-463)	250 (186-328)	130 (62-218)
	**50**	609 (468-763)	498 (391-619)	386 (304-480)	274 (208-352)	163 (94-244)
	**60**	609 (468-763)	516 (402-640)	422 (331-519)	328 (254-411)	234 (168-309)
	**70**	609 (468-763)	547 (422-675)	484 (377-593)	421 (327-518)	358 (280-444)
	**80**	609 (468-763)	578 (445-717)	546 (424-672)	514 (402-632)	483 (375-591)

© 2022 Flaxman AD et al. *JAMA Network Open.*

eReferences

1. CDC Case Surveillance Task Force. COVID-19 Case Surveillance Public Use Data [Internet]. [cited 2021 Jun 1]. Available from: https://dev.socrata.com/foundry/data.cdc.gov/vbim-akqf

2. U.S. Census Bureau. 2019 American Community Survey Public Use Microdata Samples.

3. O'Brien MP, Forleo-Neto E, Musser BJ, et al. Subcutaneous REGEN-COV Antibody Combination to Prevent Covid-19. N Engl J Med 2021;

4. Thompson HA, Mousa A, Dighe A, et al. Severe Acute Respiratory Syndrome Coronavirus 2 (SARS-CoV-2) Setting-specific Transmission Rates: A Systematic Review and Meta-analysis. Clin Infect Dis 2021;ciab100.

5. Regeneron Pharmaceuticals. REGENERON ANNOUNCES NEW U.S. GOVERNMENT AGREEMENT TO PURCHASE ADDITIONAL DOSES OF REGEN-COV™ (CASIRIVIMAB AND IMDEVIMAB) ANTIBODY COCKTAIL [Internet]. Tarrytown, New York: 2021 [cited 2022 Jan 20]. Available from: https://investor.regeneron.com/news-releases/news-release-details/regeneron-announces-new-us-government-agreement-purchase

6. Centers for Medicare & Medicaid Services. Monoclonal Antibody COVID-19 Infusion [Internet]. 2022 [cited 2022 Jan 24]. Available from: https://www.cms.gov/medicare/covid-19/monoclonal-antibody-covid-19-infusion

7. FAIR Health. Costs for a hospital stay for COVID-19 [Internet]. [cited 2021 Jul 27]. Available from: https://www.fairhealth.org/article/costs-for-a-hospital-stay-for-covid-19

© 2022 Flaxman AD et al. *JAMA Network Open.*
